# Intrathoracic Gastric Volvulus presenting with GIT Bleed

**DOI:** 10.21699/jns.v6i2.489

**Published:** 2017-04-15

**Authors:** Rahul Kadam, VSV Prasad

**Affiliations:** Department of Neonatology, Lotus Hospital for Women and Children, Hyderabad.

**Keywords:** Congenital diaphragmatic eventration, Intrathoracic gastric volvulus, Neonate

## Abstract

Intrathoracic gastric volvulus in neonatal period is a life-threatening surgical emergency. We report a case of neonate with respiratory distress and GI bleeding who was diagnosed to have congenital diaphragmatic eventration with Intrathoracic gastric volvulus.

## Case report:

A 3.1 Kg, male infant, born to 25 years old primigravida mother at 40 weeks of gestation. He was noted to have severe respiratory distress soon after birth for which he was transported to our hospital for further management. At admission, he was sick with tachycardia, tachypnea and central cyanosis. His SpO2 was 60% with 6L/minute of 100% oxygen. There was diminished air entry on left hemithorax and his abdomen was scaphoid. Emergency endotracheal intubation was performed and he was commenced on ventilatory support. Ventilatory settings were adjusted to meet adequate oxygenation. The possibilities considered were congenital diaphragmatic hernia, pneumonia, congenital lung malformations and congenital heart disease. Nasogastric tube reached the stomach without any difficulty. 

**Figure 1:**
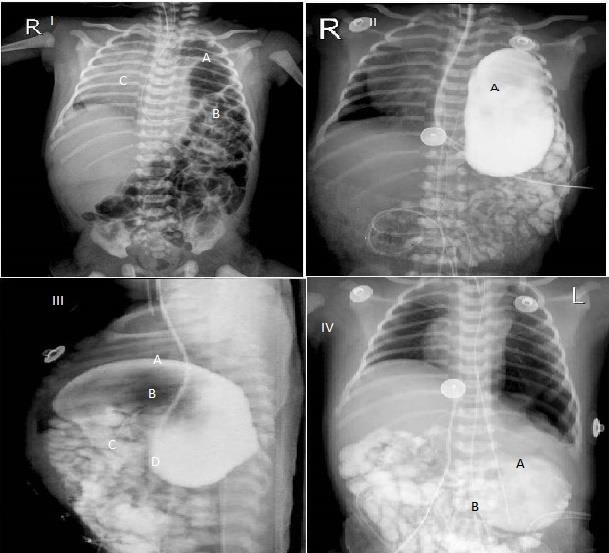
Plain CXR showing left sided congenital diaphragmatic eventration ( A) with herniation of stomach and small bowel loops (B) and mediastinal shift towards right side (C)(I). Upper GI Gastrograffin study shows the stomach and bowel loops located in the left lower chest (II-A) with greater curvature(A) noted above the lesser curvature(B) and pyloro-duodenal junction(C) noted above the gastro-esophageal junction(D) (III) with free flow of contrast from stomach. Skiagram on post-operative day 1(IV).

**Figure 2:**
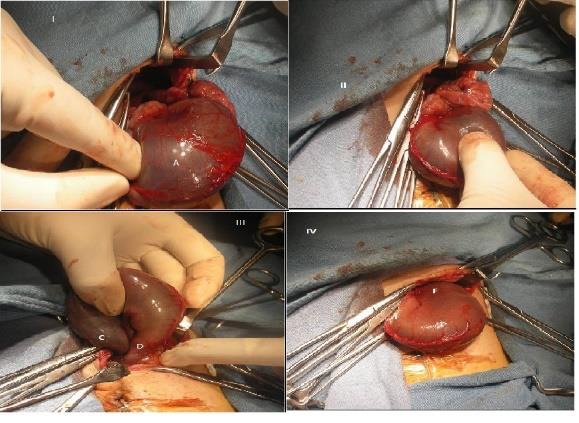
Intra-operative images showing closed loop obstruction with distended and congested stomach (I-A)) with small bowel herniating above the stomach (II-B). Mesentro-axial type of volvulus just prior to detorsion (III) with gastro-esophageal junction(C) on right side and pyloro-duodenal junction on left side (D). Post-detorsion, (IV) normal anatomy being restored with reduced distension and congestion.

He was started on parenteral nutrition and IV antibiotics following performing work-up for sepsis. His CBC at admission revealed leucocytosis. His CRP test was positive. His blood culture collected at the time of admission remained sterile. His metabolic parameters and coagulation profile were normal. His chest skiagram revealed congenital eventration of left diaphragm, gas distended bowel loops in the left hemithorax with a shift of mediastinum to the right and over distended stomach, with suspicion of gastric volvulus (Fig.1). The baby deteriorated with bleeding from nasogastric tube and frank bleeding per rectum on day 3 of admission. Possibilities considered were sepsis, coagulopathy and gastric ischemia. His coagulation profile was deranged and there was fall in hematocrit which was treated with FFP and PRBC transfusion. There was discoloration of left hemithorax. Gastrograffin study was performed which revealed mesentro-axial type of gastric volvulus in left hemithorax (Fig.1). He underwent emergency surgery through left subcostal incision. Intra-operatively there was a mesentro-axial type of gastric volvulus with distension and congestion of stomach (Fig.2). Detorsion of stomach, plication of left dome of diaphragm and anterior gastropexy was performed. He tolerated the procedure well without any intra and post-operative complications. He was gradually weaned off ventilator and extubated to oxygen by prongs on day 14 of life. He further required oxygen supplementation for 4 days, after which he could be weaned to room air. Orogastric tube feeds were initiated on day 18 of life and were graded up as tolerated by the infant. Direct breast feeds were initiated on day 26 of life and have been accepted by the infant satisfactorily thereafter. At discharge his surgical wound had healed well. His clinical physical examination was within normal limits. He is on regular follow up and doing well with normal growth parameters and development.

## Discussion: 

Intrathoracic gastric volvulus in the newborn is a rare surgical emergency. It occurs when the stomach or a part of it rotates more than 180 degrees. The stomach is relatively fixed at the esophageal hiatus and the pylorus by various ligaments. The stretching of these ligaments or their absence can cause a volvulus [1]. Because of elevated left hemidiaphragm, wide subdiaphragmatic area provides the adequate space for malrotation of the stomach, resulting in gastric volvulus [2]. Etiology of gastric volvulus is either primary or secondary. In primary cause, there is absence of diaphragmatic defects or intra-abdominal abnormality causing the volvulus. In 30% of gastric volvuli, there is a primary cause [3]. Causes of secondary gastric volvulus include congenital or traumatic diaphragmatic hernias, abdominal bands or adhesions, hiatal hernias or diaphragmatic eventration as in our case [4]. Gastric volvulus is of two types: organoaxial and mesenteroaxial. Mesenteroaxial volvulus with diaphragmatic eventration is very rare association which was there in our case. Mortality rate in acute gastric volvulus is as high as 42–56%, secondary to gastric ischemia, perforation or necrosis [4]. 

A plain abdominal x-ray and an upper gastrointestinal contrast study are useful for diagnosis. Some studies suggest that either CT or MRI are necessary for the correct diagnosis [5] but in our experience if the diagnosis is clear with plain X-ray contrast studies, surgical treatment should not be delayed with further complementary imaging tests. The standard treatment for volvulus is open laparotomy with detorsion and prevention with anterior gastropexy [4], as performed in our case. 

To conclude, congenital diaphragmatic eventration associated with gastric volvulus leads to serious complications with very high morbidity and mortality. It should be considered in diagnosis of any newborn with GI bleed. An upper gastrointestinal contrast study is useful for early diagnosis and surgical treatment should not be delayed awaiting further complementary imaging tests. 

## Footnotes

**Source of Support:** None

**Conflict of Interest:** None

## References

[1] Ascherman SW, Bednarz WW, Olix NL (1958). Gastric volvulus. Arch Surg.

[2] Par W, Choi S, Suh S (1992). Pediatric gastric volvulus: experience with 7 cases. J Korean Med Sci.

[3] And?ran F, Tanyel FC, Balkanc? F, Hiçsönmez A (1995). Acute abdomen due to gastric volvulus: Diagnostic value of a single plain radiograph. Pediatr Radiol.

[4] Chau B, Dufel S (2007). Gastric volvulus. Emerg Med J.

[5] Karabulut R, Türkyilmaz Z, Sönmez K, Karakus SC, Basaklar AC (2009). Delayed presentation of congenital diaphragmatic hernia with intrathoracic gastric volvulus. World J Pediatr.

